# Relationship between career maturity, psychological separation, and occupational self-efficacy of postgraduates: moderating effect of registered residence type

**DOI:** 10.1186/s40359-023-01261-9

**Published:** 2023-10-20

**Authors:** Jianchao Ni, Jiawen Zhang, Yumei Wang, Dongchen Li, Chunmei Chen

**Affiliations:** 1 Institute of Education & School of Aerospace Engineering, Xiamen University, Xiamen, 361005 Fujian China; 2 Silliman University, 6200 Dumaguete City, Negros Oriental Philippines; 3 Xiamen Institute of Software Technology, Xiamen, 361024 Fujian China; 4 Institute of Education, Xiamen University, Xiamen, 361005 Fujian China; 5 National Immigration Administration, Beijing, 100741 China; 6 Teachers College, Jimei University, Xiamen, Fujian 361021 China

**Keywords:** Career maturity, Psychological separation, Occupational self-efficacy, Postgraduates, Registered residence type, Mediating effect, Moderating effect

## Abstract

**Background:**

With the slowdown of economic growth and the increasing pressure of employment competition worldwide during the normalized epidemic prevention and control, the job-hunting intention and behavior of college graduates deserve in-depth study. This study explores the relationship between the career maturity, psychological separation and occupational self-efficacy of postgraduates, and provides a theoretical basis for improving their career maturity.

**Methods:**

A questionnaire survey was carried out on postgraduates with 584 valid data in China by using the Career Maturity Scale, Psychological Separation Scale and the Occupational Self-efficacy Scale. A structural equation model and bias-corrected self-sampling method were adopted to explore their relationship. The moderating effect of registered residence type was tested.

**Results:**

The results show that: (1) The higher the level of psychological separation of postgraduates, the higher their career maturity. (2) Occupational self-efficacy plays a mediating role in the process of psychological separation promoting career maturity. (3) The registered residence type moderates the latter half of the mediating process of psychological separation, occupational self-efficacy, and career maturity. Moreover, occupational self-efficacy plays a more significant role in promoting the career maturity of postgraduates with rural registered residence.

**Conclusions:**

This study reveals the relationship between the career maturity, psychological separation and occupational self-efficacy of postgraduates. At the same time, it also verifies the mediating role of occupational self-efficacy and the moderating role of registered residence type. The result is helpful for postgraduates to understand the level of their career maturity and improve their career decision-making level and career development ability.

## Introduction

The employment of college graduates is related to people’s well-being, social stability and high-quality development. It is also an important indicator to measure the quality of talent training in colleges and universities. Career maturity, initially proposed by career guidance expert Super in the 1950s (Super, 1953), is a crucial evaluative indicator for assessing individual career development [1]. He defines career maturity as the psychological, social and physical readiness of young people to choose a career (Super, 1981) [2]. Building upon Super’s research, Crites (1978) puts forward a more mature theory on the basis of Super’s research [3]. He holds that career maturity could be used to represent the degree of individual career development and the state of preparation for making career choices. Its key factor is to have clear, rational and correct career goal design and career planning. Furthermore, Kleine et al. (2021) emphasize that career maturity refers to the ability to independently and responsibly make career decisions based on integrating oneself and the work environment [4]. In this view, career maturity represents not only the individual’s preparedness to choose a career but also their capacity to navigate and adapt to the complexities and dynamics of the work world. By integrating Super’s foundational work, Crites’ emphasis on career development, and Kleine expanded perspective on decision-making and integration, a comprehensive understanding of career maturity emerges. It encompasses the psychological, social, and physical readiness to select a career, the level of an individual's career development and preparedness for decision-making, and the ability to make autonomous and responsible career choices while integrating oneself with the work environment.

Various scholars have conducted in-depth research on career maturity. Scholars find that career maturity has a great impact on the selection of individual positions, and it is the key factor to measure college students’ employment success (Ju & Shin, 2020; Zhang et al., 2018) [5, 6]. Tong Huijie’ (2013) studies show that career maturity could predict the probability of an individual successfully obtaining a position [7]. Moreover, it could effectively predict the job adaptation and job performance of newly recruited college students. The higher the career maturity, the easier it is for an individual to make a suitable career choice, which is correspondingly more conducive to the individual’s career success (Liu Hongxia, 2009) [8]. In addition, research has supported the idea that self-concept seems to have an effect on career maturity (Greenhaus, 1971) [9]. Helbing (1984) holds that career maturity is correlated with work orientation and a sense of personal identity [10]. Dillard (1976) indicates that the relationships between career maturity and self-concepts are relatively weak-positive [11]. Shelley (1977) concludes that as to the relationship between self-concept, self-actualization and career maturity, a positive self-concept is necessary [12].

At present, the research on career maturity mainly focuses on undergraduates. There is little research on graduate students, especially postgraduates. Compared with undergraduates, postgraduates have different psychological development and major contradiction in life. They have received a deeper level of higher education and more systematic learning and understanding of professional knowledge. Therefore, the structure, as well as the development characteristics of their career maturity might be different. Therefore, the career maturity of this group has potential value for further research. According to the “National Statistical Bulletin on the Development of Education” issued by China’s Ministry of Education, the country’s graduate enrollment has risen year by year in the past three years. Therefore, the scarcity and competitive advantage of a master’s degree in the job market is gradually decreasing, and the employment pressure they face is gradually increasing. Nowadays, the overall employment market of postgraduates has presented problems such as the mismatch between professional ability and quality with the requirements of employers, mismatch of disciplines and majors with emerging industries, unsynchronized job search and recruitment of employers, and gaps between traditional employment concepts and employment requirements in the new era (Li Jian, 2020) [13]. Some postgraduates have high expectations of salary and benefits, and are easily affected by the psychological impact of “Being unfit for a higher post but unwilling to take a lower one”, and their professional ability and as well as self-evaluation are prone to deviations, that is, some postgraduates have not yet reached their due level of career maturity. Therefore, it is important to explore the characteristics and influencing factors of postgraduates’ career maturity.

The factors that affect individual career maturity mainly include individual psychology factors (such as psychological separation, career efficacy, etc.) (Lv Aiqin et al., 2008) [14], family background factors (such as parental occupation type, family economic status, etc.) (Sun & You, 2019; Puebla, 2022) [15, 16] and social characteristics factors (such as gender, age, registered residence, etc.) (Bae, 2017; Park & Jun, 2017) [17, 18]. Lee & Hughey (2001) hold that a large part of the healthy development of occupation depends on the degree of psychological separation between individuals and their parents [19]. They believe that psychological separation has an important impact on career maturity. Patton et al. (2005) hold that career efficacy might affect career maturity [20]. Then, how does psychological separation affect career maturity through occupational self-efficacy? To this end, this study introduced the variable related to family factors “psychological separation”, the variable related to individual psychological factors “occupational self-efficacy”, the variable related to social characteristics factors “registered residence type”. Postgraduates are selected as the research object, and career maturity is taken as an indicator to measure the willingness and ability of personal career development. The mechanism of psychological separation, and occupational self-efficacy on postgraduates’ career maturity was discussed. The mediating effect of occupational self-efficacy and the moderating effect of registered residence registration type were clarified.

## Theoretical hypotheses

### Career maturity, psychological separation, and occupational self-efficacy

Super formally put forward the concept of career maturity. Career maturity is now at the center of career counseling and education programs in various schools, as well as being incorporated into many business, industry, and government career development programs. Career maturity is also the most commonly used outcome measure in career counseling and is widely used internationally. With the continuous development of career maturity theory, multinational researchers led by Crites, Savickas and Westbrook have discussed career maturity from different perspectives and accumulated a series of research results. Although there are various categories of career maturity nowadays, it can be generally classified into the following three points: Firstly, almost all scholars recognize the dynamic nature of career development and believe that it is a process of continuous development and advancement. Secondly, scholars’ definition of career maturity pays attention to the role of individual cognitive ability on career maturity. Third, the definition of career maturity emphasizes the individual's subjective initiative in the process of career development. This study is based on Crites’ definition of career maturity. Crites comprehensively summarizes the structure of career maturity and helps people understand their stage and development tasks in the career development process. Individuals’ career maturity is closely related to the family environment. Vondracek et al. (1986) hold that if there is variable that can predict an individual’s occupational status, this variable is the socioeconomic status of the individual’s original family [21]. Individuals’ career maturity is not only affected by the intergenerational transmission of family status, but also by the relationship with parents. The degree of individual separation from parental dependence, that is, psychological separation, will significantly affect their career maturity, which in turn affects their occupational development level (Lee & Hughey, 2001) [19]. The higher the degree of separation between adolescents and their original families, the higher the individuals’ sense of professional competence (Frank et al., 1988) [22]. Son Hyun Sook (2009) explores the relationship between career maturity and psychological separation [23]. He finds that the higher the level of individual maternal psychological separation, the higher the level of career maturity. Accordingly, this study proposes the following hypotheses:

- **Hypothesis H1: **The psychological separation of postgraduates is positively related to their career maturity.

Individuals’ career maturity is also affected by individuals’ career psychological factors, of which the influence of occupational self-efficacy is particularly prominent (Hazel, 2022) [24]. Bandura (1977) proposes a theory of self-efficacy to explain the reasons for people’s motivation in certain situations [25]. Spencer & Bandura (1987) believe that self-efficacy is individuals’ assessment of the degree of confidence in one's ability to complete a task. The results of the assessment will affect their subsequent motivations and choices [26]. Taylor & Betz (1983) propose career decision-making self-efficacy on the basis of Bandura’s self-efficacy theory [27]. They believe that career decision-making self-efficacy refers to the self-evaluation or confidence of decision-makers in the process of career decision-making in their ability to complete various tasks. Hou Chunna et al. (2013) show that sound and independent personality development (such as a sense of responsibility) would affect the development level of individual college students. This makes them have higher self-efficacy, which could be reflected in the face of career decision-making for occupational self-efficacy [28]. The “Social Cognitive Career Theory” proposed by Song & Chon (2012) suggests that the career maturity of individuals might be related to their occupational self-efficacy [29]. Individuals with high occupational self-efficacy tend to have positive expectations for their career development. This positive expectation will drive them to take measures to meet various challenges in their career. Individuals with high occupational self-efficacy have clearer career goals and are more active in exploring career self, career information and career planning (Du Rui, 2006) [30]. Empirical research also proves that occupational self-efficacy is positively related to career maturity (Abdullah, 2023) [31]. YongHee & HyunSoon (2019) show that self-encouragement and occupational self-efficacy play a complete mediating role in the relationship between adolescent peer attachment and career maturity [32]. Kim Daeyoung & Joeng Ju ri. (2018) show the mediating role of career decision-making self-efficacy between parents’ active learning participation and career maturity [33]. Zhang Hua (2008) shows that the success of the psychological separation process would also help individuals to establish independence and autonomy, and get rid of parental attachment [34]. Therefore, individuals are more likely to obtain successful experiences, a positive psychological state, a positive external environment of trust, resulting in strong self-efficacy. The positive attitude of actively pursuing success is conducive to the development of a higher sense of career choice efficacy (Ye Baojuan et al., 2020) [35]. The success of the individualization process after psychological separation is conducive to the formation of healthy occupational psychology. Positive occupational psychology is also the driving force for career maturity. Good occupational self-efficacy plays a catalytic role. Combined with the above discussion, we infer that occupational self-efficacy might be the key factor linking the psychological separation and career maturity of postgraduates. Accordingly, this study proposes the following hypotheses:

- **Hypothesis H2:** Occupational self-efficacy mediates the positive relationship between psychological separation and career maturity.

### The moderating effect of registered residence type

In most countries, registered residence registration is mainly used to register the change of residence. The difference between urban and rural registered residence only exists as the difference of residence. The registered residence system is unique in China, since China implements a dual registered residence system in urban and rural areas, which links individual registered residence with specific regions, and divides registered residence into urban and rural types. The original purpose of the system was to restrict the cross-regional mobility of residents. In the long-term development, the residence registration system not only plays the role of registered residence management, but also affects various aspects of society, such as the occupation, medical care, education and social security of residents (Li Zhenjing&Zhang Linshan, 2014) [36]. The differences of registered residence have different impacts on public resources and social welfare (Jiancai Pi & Pengqing Zhang, 2016) [37]. With the rapid development of the economy in China, there are more differences in economic conditions and resource allocation among different regions. Due to the different household registered residence types of individuals, the external differences in the region tend to have influence on their career awareness, career knowledge and career attitude. Therefore, individual career maturity might have different performances in urban and rural samples. The research results around the differences in career maturity between urban and rural areas have drawn different conclusions. Chinese scholars Jia Pengfei & Chen Zhenbang (2011) find that there is no significant difference between urban and rural sources of college students’ career maturity [38]. Scholars in other counties generally believe that the career maturity of students is affected by their registered residence. Research by Alam (2016) shows that there are significant differences in career maturity between rural and urban students [39]. Junga & Yuntae (2015) find that adolescents living in cities tend to have higher career maturity. Adolescents living in rural areas have lower career maturity due to a lack of relevant social support [40]. Vibha & Ushakiran (2016) measure the career maturity of adolescents and find that the career maturity score of urban adolescents is higher than that of rural samples [41]. In addition, there are differences between urban and rural areas in individual occupational self-efficacy. The research of Conceicao et al. (2016) shows that there are significant differences between rural and urban teachers’ occupational self-efficacy [42]. Casapulla (2017) finds that in the process of participating in urban and rural services, students’ self-efficacy in providing vocational services in urban and rural areas is affected by their type of residence [43]. Students in different places of residence showed different self-efficacy in this process. To sum up, we believe that differences in registered residence attributes bring about different performances of postgraduates’ occupational self-efficacy and career maturity. The relationship between postgraduates’ occupational self-efficacy and career maturity is affected by registered residence attributes. Accordingly, this study proposes the following hypotheses:

- **Hypothesis H3:** Registered residence type moderates the positive relationship between occupational self-efficacy and career maturity, and such relationship is stronger in rural registered residence rather than in urban registered residence.

**Fig. 1:**
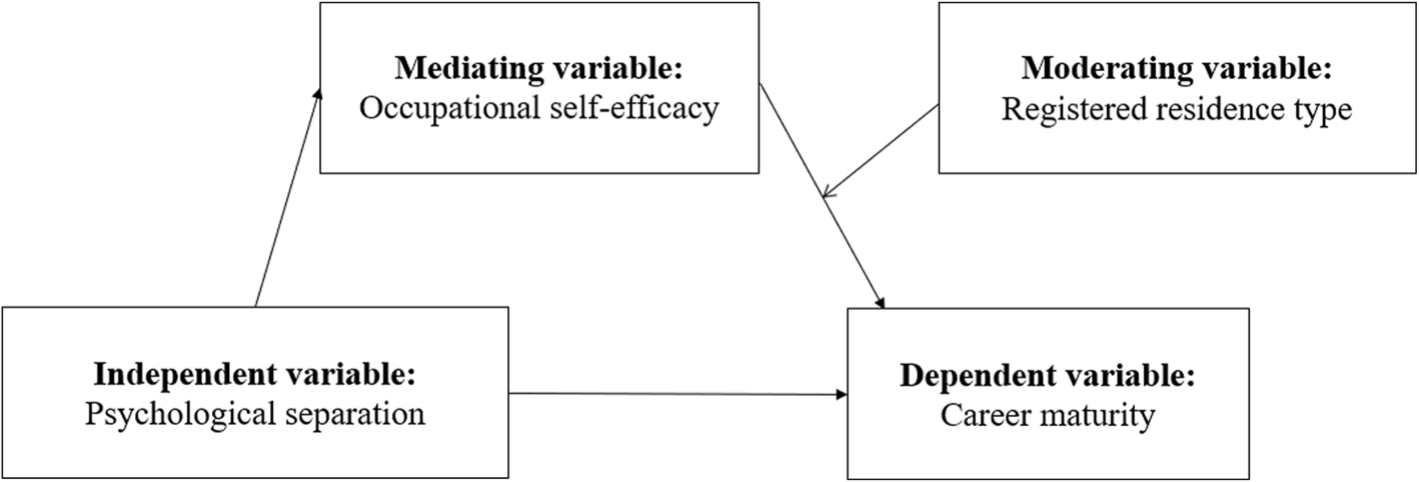
Hypothetical model of the mediating effect of occupational self-efficacy and the moderating effect of registered residence type

To sum up, this study aims to study the relationship between the career maturity, psychological separation and occupational self-efficacy of postgraduates, and examine the mediating role of occupational self-efficacy and the moderating role of the registered residence type. The hypothesis model is shown in Fig. 1.

## Method

### Data collection

On the basis of ensuring the scientific design of the survey, considering the feasibility of the design and the principle of economy and effectiveness, this study adopts the convenient sampling method. Samples are taken from easily available subjects. This method is fast, simple, easy to obtain and cost-effective (Henry, 1990) [44]. By this method, a survey of postgraduates from different regions and levels of universities in China such as Xiamen University, Guangxi University (the source universities are widely distributed) was carried out and 600 questionnaires were collected by our research team. The questionnaire was mainly a paper version, supplemented by an electronic version, and data were collected synchronously through a combination of online and offline. The online questionnaire is distributed by using the website of Wenjuanxing (https://www.wjx.cn/) to forward the questionnaire to the group and invite students to fill in the form of red packets. The offline questionnaire is distributed by giving small gifts to students. After sorting, 584 valid questionnaires were finally obtained, 16 invalid questionnaires with characteristics such as short filling time, missing data, and suspected insincere answers were excluded, and the effective recovery rate of the questionnaire was 97.33%. The study is only a questionnaire survey and does not involve human clinical trials or animal experiments, which conforms to ethical standards.

### Research tools

#### Career Maturity Scale

The master graduates’ career maturity questionnaire was used to measure the postgraduates’ career maturity (Wang Yumei, 2020) [45]. 18 typical items (with the sample items such as: “I know what kind of work I like”, “I can describe the main work contents of the occupation I am interested in” etc.) were selected to measure the career choice ability, career choice attitude, career choice knowledge of postgraduates, aiming to reflect the various abilities of postgraduates in the process of career choice. The 18 questions were graded on a 4-point scale: "1 = very inconsistent", "2 = relatively inconsistent", "3 = relatively consistent", and "4 = very consistent". The higher the score, the higher the degree of agreement. Confirmatory Factor Analysis (CFA) was performed on the scale, and the fitting index parameters were obtained as follows: χ^2^/df (ratio of chi-square to degrees of freedom) = 2.361, CFI (Comparative Fit Index) = 0.972, IFI (Incremental Fit Index) = 0.972, GFI (Goodness of Fit Index) = 0.952, RMSEA (Root Mean Square Error of Approximation) = 0.048. The Cronbach’s α coefficient of the scale was 0.943, which showed good consistency and the measurement results were valid. The 18 items were summed up and averaged to obtain the variable of career maturity, which was used to represent the career maturity of postgraduates. The higher the score, the higher the degree of career maturity.

#### Psychological separation scale

The psychological separation questionnaire was used to measure the degree of psychological separation of postgraduates (Wu Huiqing, 2012) [46]. This questionnaire has been used and proved to be effective. Although this questionnaire is designed for undergraduates’ psychological separation, psychological separation is a relatively stable concept, which is applicable to people of different ages and different educational levels. Both undergraduates and postgraduates are in a relatively similar environment, facing similar pressures and challenges. And currently, there are no other psychological separation scales more suitable for postgraduates. Therefore, this questionnaire still has reference value in measuring the psychological separation of postgraduates. In a practical study, selecting all the items might lead to the scale being too long, increasing the time, cognitive burden and discomfort of the subjects, thus affecting the quality of their responses. Some questions are less difficult or lack differentiation. Therefore, on the premise of ensuring the reliability and validity of the measurement, eliminating some items can improve the differentiation of the scale. In this study, a total of 8 typical items (with the sample items such as: “I feel especially in need of comfort when I encounter setbacks”, “I seek support when making decisions or plans” etc.) were selected from emotion separation, attitude separation and behavior separation respectively, and the corresponding items could effectively reflect the concept to be measured, help to improve the validity of the scale and reduce measurement errors. Each question adopted a 4-point scale: "1 = very inconsistent", "2 = relatively inconsistent", "3 = relatively consistent", and "4 = very consistent". The higher the score, the higher the degree of agreement. The CFA was performed on the scale, and the parameters were obtained as follows: χ^2^/df = 1.419, CFI = 0.997, IFI = 0.997, GFI = 0.993, RMSEA = 0.027. The Cronbach’s α coefficient of the scale was 0.869, which showed good consistency and the measurement results were valid. The 8 items were summed up and averaged to obtain the variable of psychological separation, which was used to represent the degree of psychological separation of postgraduates. The higher the score, the higher the degree of psychological separation.

#### Occupational self-efficacy scale

The occupational self-efficacy scale was used to measure the occupational self-efficacy of postgraduates (Schyns & Collani, 2002) [47]. According to the actual situation of postgraduates in China, 9 typical items (with the sample items such as: “I have a way of getting what I want even if others are against me”, “It's easy for me to stick to my ideals and achieve my goals” etc.) were finally retained to measure the degree of self-confidence of postgraduates in completing corresponding professional behaviors and achieving career goals. The 9 questions were graded on a 4-point scale: "1 = very inconsistent", "2 = relatively inconsistent", "3 = relatively consistent", and "4 = very consistent". The higher the score, the higher the degree of agreement. The CFA was performed on the scale, and the parameters were obtained as follows: χ^2^/df = 1.345, CFI = 0.998, IFI = 0.998, GFI = 0.994, RMSEA = 0.024. The Cronbach’s α coefficient of the scale was 0.901, which showed good consistency and the measurement results were valid. The 9 items were summed up and averaged to obtain the variable of occupational self-efficacy, which was used to represent the degree of the occupational self-efficacy of postgraduates. The higher the score, the higher the degree of occupational self-efficacy.

### Statistical analysis

Consideration was given to the possibility of common method bias arising from the use of self-report data collection. Therefore, the procedure of this study was controlled by an anonymous survey and reverse scoring of some questions. At the same time, Harman’s single-factor test was used to test the data for common method bias. The results showed that there were 4 factors with eigenvalues greater than 1, and the total variance explained by the first common factor was 35.63%, which was less than the critical value of 40%. Therefore, the data in this study did not have the problem of common method bias (Zhou Hao&Long Lirong, 2004) [48]. SPSS26.0 was used for reliability analysis, confirmatory factor analysis and correlation analysis. The macro PROCESS of SPSS procedure was used to test the hypothesis of the moderated mediation model.

## Research results

The basic characteristics of the sample are shown in Table 1. The distribution of samples in demographic variables is relatively balanced, showing good representativeness.

**Table 1:** Demographic characteristics (*N* = 584)

Variables	Gender		Major				Only child		Residence		
	Male	Female	Science	Engineering	Liberal arts	Others disciplines	Yes	No	Urban	Rural	Total
Percentage	58	42	19.5	53.1	24	3.4	42.8	57.2	48.1	51.9	100

### Correlation analysis between main variables

Correlation analysis was carried out on the main variables for the career maturity, occupational self-efficacy and psychological separation. Considering that the main variables were continuous, the Pearson correlation coefficient test was used. The results in Table 2 showed that the psychological separation, occupational self-efficacy and career maturity were positively correlated. Psychological separation was positively correlated with occupational self-efficacy (*r* = 0.26, *p* < 0.01), and positively correlated with career maturity (*r* = 0.19, *p* < 0.01). Occupational self-efficacy was positively correlated with career maturity (*r* = 0.67, *p* < 0.01).

**Table 2:** Descriptive statistics and correlation coefficient matrix of each variable

Variable	M	SD	1	2	3	4	5	6	7
1. Psychological separation	2.55	0.58	1						
2. Occupational self-efficacy	2.87	0.50	0.26 ^**^	1					
3. Career maturity	3.04	0.45	0.19 ^**^	0.67 ^**^	1				
4. Gender	0.58	0.49	-0.07	0.09 ^*^	0.02	1			
5. Major	0.27	0.45	-0.16 ^**^	-0.12 ^**^	-0.05	-0.38 ^**^	1		
6. Whether the only child	0.43	0.50	0.07	-0.01	-0.06	0.02	-0.05	1	
7. Registered residence type	0.48	0.50	0.01	-0.01	0.02	-0.04	0.09 ^*^	0.40 ^**^	1

*Note*: *:*p* < 0.05, **:*p* < 0.01, ***:*p* < 0.001 (The same as below)

### The relationship between psychological separation and career maturity: a moderated mediation test

The test of the research hypothesis refers to the procedure of Wen Zhonglin’s moderated mediation test (Wen Zhonglin, 2014) [49]. The mediation model is constructed and the moderating variables are introduced to explore the model. According to this judgment standard, this section examines the moderating effect of registered residence type on the mediating process of "psychological separation, occupational self-efficacy, and career maturity". Control variables such as major, whether the only child and gender type were virtualized.

Firstly, the mediating effect of occupational self-efficacy between psychological separation and career maturity was tested under the control of major, whether the only child and gender type. The specific results were shown in Table 3 below. The results showed that psychological separation had a significant promoting effect on career maturity (β = 0.195, t = 4.721, *p* < 0.001), which passed the 99.9% significance level test, Therefore, hypothesis 1 was supported. Psychological separation also had a promoting effect on occupational self-efficacy (β = 0.26, t = 6.410, *p* < 0.001), which also passed the 99.9% significance level test. Referring to the idea of Wen Zhonglin’s mediation effect test, it can be considered that occupational self-efficacy played a mediating role between psychological separation and career maturity. It was a complete mediator, that was, the proportion of the mediation effect was 100%. Therefore, occupational self-efficacy mediated the relationship between psychological separation and career maturity, and Hypothesis 2 was supported.

**Table 3:** The mediating effect of occupational self-efficacy

Variable	**Equation 1**			**Equation 2**			**Equation 3**		
	Career maturity			Occupational self-efficacy			Career maturity		
	SE	β	t	SE	β	t	SE	β	t
Psychological separation	0.041	0.195 ***	4.721	0.040	0.26***	6.410	0.032	0.022	0.665
Occupational self-efficacy							0.032	0.669 ***	20.855
Gender	0.090	0.025	0.574	0.088	0.087*	2.010	0.068	-0.033	-0.980
Major	0.100	-0.020	-0.440	0.098	-0.058	-1.336	0.076	0.019	0.575
Whether the only child	0.082	-0.077	-1.896	0.081	-0.032	-0.791	0.062	-0.056	-1.822
R square	0.037			0.076			0.449		
F	6.553***			12.942***			96.156***		

*Note*: β is the standardized coefficient; R square is the adjusted R square

Secondly, in order to further verify the mediating effect of occupational self-efficacy, the bootstrap sampling method (1000 times of sampling) was adopted to obtain the bootstrap test results of the mediating effect, which were arranged in Table 4 below. The results showed that the 95% confidence interval of the indirect effect did not contain 0, and the 95% confidence interval of the direct effect contained 0, that was, the indirect effect existed, the direct effect did not exist, and the occupational self-efficacy played a complete mediating role.

**Table 4:** Decomposition table of indirect effect, direct effect and mediation effect

Effect name	Effect size	Boot standard error	95%CI	Mediation ratio
Indirect effect	0.174	0.036	(0.102, 0.242)	100%
Direct effect	0.022	0.032	(-0.042, 0.085)	

Again, model 14 in the macro PROCESS of SPSS plug-in compiled by Hayes (2012) (model 14 assumes that the second half of the indirect effect in the mediation model is moderated, which is in line with our hypothesis expectations) is used. Hayes developed the plugin PROCESS based on SPSS for mediating and moderating effect analysis. Process is a plug-in that specializes in mediating and moderating effects analysis, providing more than 70 models, the analysis process needs to select the corresponding model, set the corresponding independent variables, dependent variables, mediating or moderating variables, which can facilitate the operation and analysis of mediation models, moderated mediation models, etc. The moderating effect of registered residence type was tested under the control of major, whether the only child and gender, and the following Table 5 was obtained. The results showed that psychological separation significantly promoted occupational self-efficacy (β = 0.260, t = 6.410, *p* < 0.001), and occupational self-efficacy significantly promoted career maturity (β = 0.771, t = 17.655, *p* < 0.001). The complete mediation of occupational self-efficacy between psychological separation and career maturity was still valid. There was a positive relationship between registered residence type and career maturity (β = 0.113, t = 1.696, *p* < 0.1), and the career maturity of postgraduates with rural registered residence was higher than that of postgraduates with urban registered residence. The interaction item of occupational self-efficacy and registered residence type was significant in the model (β = -0.209, t = -3.42, *p* < 0.001), that was, the interaction item between occupational self-efficacy and registered residence type could significantly affect career maturity. Therefore, registered residence type moderated the relationship between occupational self-efficacy and career maturity.

**Table 5:** Test of the moderated mediating effect of psychological separation on career maturity

Variable	**Equation 2**			**Equation 3**		
	Occupational self-efficacy			Career maturity		
	SE	β	t	SE	β	t
Psychological separation	0.040	0.260***	6.410	0.032	0.014	0.443
Occupational self-efficacy				0.044	0.771***	17.655
Registered residence type				0.067	0.113!	1.696
Occupational self-efficacy × Registered residence type				0.061	-0.209***	-3.420
Gender	0.088	0.087*	2.010	0.067	-0.072	-1.062
Major	0.098	-0.058	-1.336	0.075	0.023	0.303
Whether the only child	0.081	-0.032	-0.791	0.068	-0.156	-2.311
R square	0.076			0.468		
F	12.942***			72.278***		

**Fig. 2:**
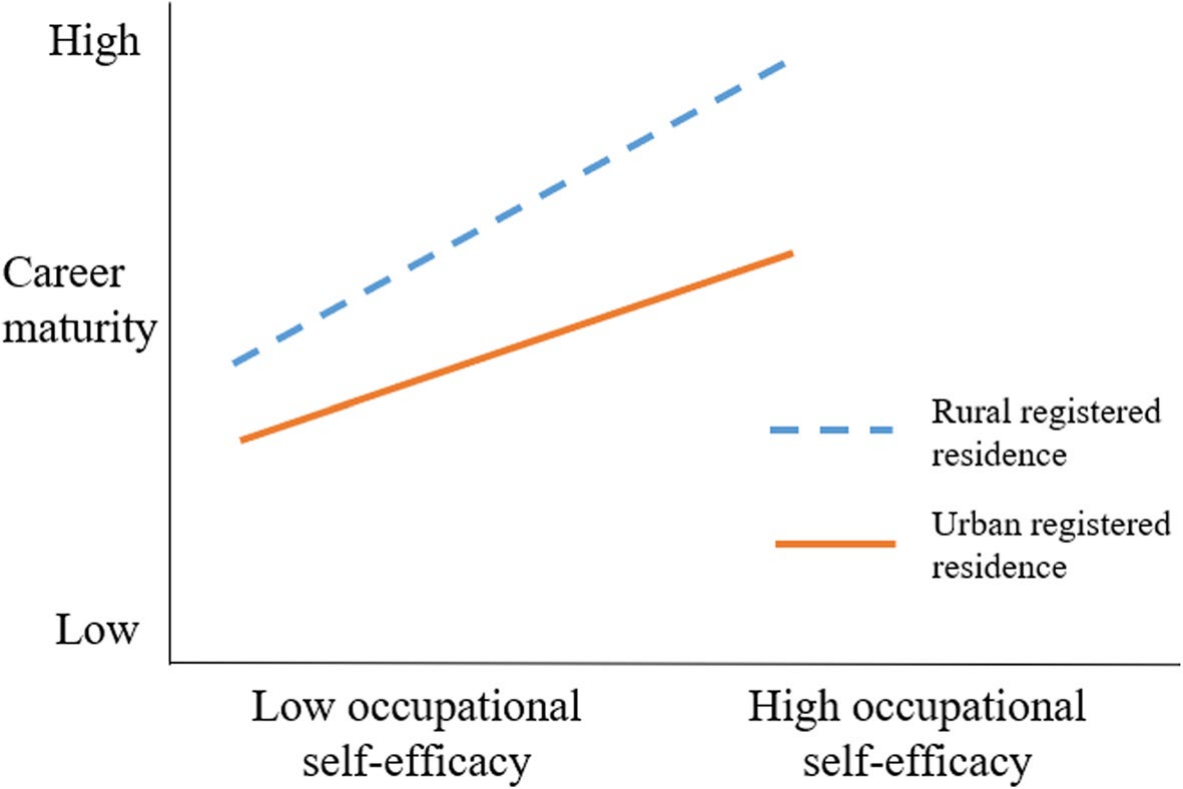
The moderating effect of registered residence type on occupational self-efficacy and career maturity

Finally, in order to more vividly interpret the moderating effect of registered residence type on occupational self-efficacy and career maturity, the research subjects were grouped according to registered residence type. A simple slope test was performed to obtain Fig. 2 below. The results showed that occupational self-efficacy played a positive role in promoting the career maturity of postgraduates (all slopes were greater than 0). In general, at all stages of occupational self-efficacy, the career maturity of postgraduates with rural registered residence was higher than that of postgraduates with urban registered residence. With the rise of occupational self-efficacy, the career maturity of postgraduates with rural registered residence increased faster. In fact, the rate of career maturity improvement of postgraduates with urban registered residence was slower than that of postgraduates with rural registered residence. This indicates that the occupational self-efficacy of postgraduates with rural registered residence had a stronger role in promoting their career maturity. Therefore, Hypothesis 3 was supported.

## Discussion

In this study, we constructed and tested a moderated mediation model to examine the moderating effect of registered residence type on the mediating process of “psychological separation, occupational self-efficacy, and career maturity”. The results showed that the moderating variable “registered residence type” had a significant moderating effect on the mediating path.

Firstly, the results of correlation analysis and structural equation analysis proved that the positive effect of psychological separation on career maturity was significant and robust. This is consistent with previous research results. Previous studies showed that there was a close relationship between psychological separation and career maturity. Lopez & Andrews (2014) found that the degree of separation between children and families had a significant impact on individuals’ career decision-making, and a better level of psychological separation might reduce the difficulty of individuals’ career decision-making [50]. Zhang Xinyong et al. (2014) pointed out in their research that the more able an individual was to take responsibility in the event of a conflict with parents, the higher their level of career maturity [51]. Therefore, college educators or psychological consultants should focus on strengthening the psychological counseling work for postgraduates. Guide them to achieve emotional, attitude and behavioral independence on the premise of maintaining emotional contact with their parents, and promoting their career maturity.

Secondly, the results of the mediation effect test showed that psychological separation not only directly promoted the career maturity of postgraduates, but also promoted the occupational self-efficacy of postgraduates. Moreover, it could indirectly affect career maturity through occupational self-efficacy. Occupational self-efficacy played a completely mediating role between psychological separation and career maturity. This was consistent with the previous studies. The research of Kang & Hyun-Wook (2016) showed that psychological separation had a positive effect on individuals’ occupational self-efficacy[52]. Chen Yuaner & Ma Xiaoqin (2016) found that the two dimensions of occupational self-efficacy, occupational cognition and occupational value, had a positive role in promoting career maturity [53]. Gao Shanchuan & Sun Shijin (2005) found that the influence of occupational self-efficacy on individual career maturity was not only reflected in a direct role, but also played an indirect role [54]. The research of Liu Yang et al. (2022) on the career maturity of college students showed that occupational self-efficacy played a mediating role between their craftsman psychology and career maturity, that was, craftsman psychology could indirectly predict career maturity through occupational self-efficacy [55]. Psychological separation could positively promote career maturity, but there were various factors that affect career maturity, such as occupational self-efficacy, and career maturity was a dynamic change process (Betz et al., 1981) [56]. Occupational self-efficacy could help individuals achieve more positive results in the process of job hunting. Specifically, individuals with high occupational self-efficacy were more confident in achieving career goals. They could objectively and comprehensively conduct self-analysis and evaluation, and have a more objective understanding of their personality traits, abilities, interests, etc. (Li Zhengwei et al., 2010) [57]. In addition, they could position their career direction more accurately and their career goals were clearer (Ochs & Roessler, 2004) [58]. At the same time, they were usually more involved in the process of career selection, and were able to explore and learned more actively (Blustein, 1989) [59]. They collected occupational and industry information related to their career goals to have a more comprehensive understanding of professional knowledge and positions. They could timely understand and discover the needs and changes of the occupational environment (Qu Kejia et al., 2015) [60], flexibly respond to difficulties encountered in the process of career selection, and be willing to adjust the target appropriately according to the actual situation (Savickas et al., 2002) [61]. Therefore, individuals with high occupational self-efficacy would have more confidence in their careers, have more active job-seeking behaviors, have stronger career decision-making abilities, and have a higher level of career maturity. Colleges and universities could formulate scientific career planning courses according to the development law of occupational self-efficacy of postgraduates. Through cultivating good career self-efficacy of postgraduates, the career development level of postgraduates can be improved.

Finally, the moderated mediation effect test showed that registered residence type played a moderating role between occupational self-efficacy and career maturity. The results showed that the career maturity of postgraduates with rural registered residence was higher than that of postgraduates with urban registered residence, which was consistent with the research results of He Weijie et al. (2022) [62]. However, the results are inconsistent with Junga & Yuntae (2015) [40], Vibha & Ushakiran (2016) [41]. Occupational self-efficacy not only played a positive role in promoting the career maturity of postgraduates, but also the magnitude of this effect varied among postgraduates with different registered residence types. Occupational self-efficacy had a more significant role in promoting the career maturity of postgraduates with rural registered residence. With the rise of occupational self-efficacy, the career maturity of postgraduates with rural registered residence increased faster than that of postgraduates with urban registered residence. The reason might be as follows: The long-term urban–rural dual policy in China has resulted in a social and economic imbalance between rural and urban areas. Due to the influence of the economy, family cultural structure, parental education methods, etc., compared with urban registered residence postgraduates, rural registered residence postgraduates are more likely to show insufficient confidence and inferiority. This also affects the career choice of urban and rural postgraduates to some extent. For example, rural registered residence postgraduates might reduce their career opportunities, and they are less likely to enter the government organs and state-owned enterprises, engage in elite occupations and obtain high-income industries than urban registered residence postgraduates. From the perspective of social capital, this phenomenon is directly related to the lack of social capital owned by rural registered residence postgraduates. Bourdieu Pierre (1980) holds that social capital means that when a person has a certain kind of lasting relationship network, the relationship network composed of people who are familiar with each other means the resources he or she actually or potentially owns [63]. Yu Hui & Hu Zixiang (2019) believe that young people’s career choice is also influenced by strong relational social capital, especially strong relational capital of talent [64]. Individuals with better family conditions often rely on strong family social relationship capital to obtain employment opportunities. However, strong family social relationship capital will gradually develop into weak relationships over time, while individual social capital will gradually show strong relationship over time. Most urban registered residence postgraduates have better social capital than rural registered residence postgraduates, this kind of social capital is mainly provided by the previous generation, which has been a fact. For rural registered residence postgraduates, the lack of abundant social capital makes them pay more attention to the accumulation of psychological capital, so they are more independent, hard-working, especially focus on the improvement of occupational self-efficacy, that is, they do not rely too much on the power of social capital. The belief in achieving career goals is an internal drive. Those rural registered residence postgraduates who have a strong belief in achieving career goals have a stronger motivation for career exploration and more career exploration behaviors. They are more fully prepared to collect career information and formulate career goals, and dare to face challenges in career development. They have a clear grasp of their professional abilities. Career maturity will also be higher. However, urban registered residence postgraduates are generally more confident because of their relatively favorable family environment and educational conditions. Their belief in achieving career goals has a weaker influence on their career maturity level. Therefore, occupational self-efficacy has a greater role in promoting the career maturity of rural registered residence postgraduates.

As research on career maturity mainly focuses on undergraduate students, there is little research on postgraduates. This study further enriches the research field of career maturity. Based on Crites’ career maturity theory, this paper uses occupational self-efficacy to explain the mechanism of psychological separation on career maturity, clarifies the logical relationship between postgraduates’ psychological separation and career maturity, and explores the mediating effect of occupational self-efficacy and the moderating effect of registered residence type. It further supplements and enriches the relevant theoretical research on career maturity, and provides theoretical support for the targeted career planning education of postgraduates, improving and enhancing the level of postgraduates’ career development. Meanwhile, this paper still has the following limitations: Firstly, this study uses cross-sectional data, so we couldn’t see the trend of time changes, and the impact of time effects on the conclusion is ignored. Secondly, due to the limitations of the questionnaire design, the measurement of some variables might not be precise enough to cover all aspects of the relevant concepts, and there might still be omissions. Third, limited by familiarity with the relevant topics, there might be omissions in the selection of control variables, which might have a certain impact on the research conclusions. Finally, although this paper uses a robust test mechanism for mediating and moderating effects, it fails to explore the causal mechanism in-depth and lacks the identification of causal relationships. Therefore, future related research can be improved from the following aspects: Firstly, expand the scope of the study, strive to include more types of schools as the sampling frame, and select the final sample by random sampling, so as to ensure a completely random data as much as possible and avoid bias in model estimation due to sampling error. Meanwhile, strive to collect data for the same sample for multiple years, so as to control the impact of time effects on the relevant variables of the sample, and obtain a more robust and reliable research conclusion. Secondly, improve the design of the questionnaire and strive to design a more realistic scale to ensure more accurate measurement. At the same time, increase the control variables in the questionnaire to include some demographic characteristics and social structure characteristics, so as to avoid the estimation error caused by various dependent variables. Third, improve the research method and use the model that can identify the causal mechanism to discuss the relationship between the research objects, so as to ensure that the obtained regression relationship is accurate and reliable.

## Conclusions

The results show that: (1) Psychological separation has a significant positive effect on the occupational self-efficacy and career maturity of postgraduates. The higher the level of psychological separation, the higher the level of career maturity of postgraduates. Occupational self-efficacy also has a significant positive effect on the career maturity of postgraduates. (2) Occupational self-efficacy plays a complete mediating role between the psychological separation and career maturity of postgraduates. Psychological separation not only affects the career maturity of postgraduates directly, but also has an indirect effect on career maturity through occupational self-efficacy, and this indirect effect has a 100% mediating effect. (3) Registered residence type plays a moderating role between occupational self-efficacy and career maturity. Occupational self-efficacy not only has a positive predictive effect on the career maturity of postgraduates, but also the magnitude of this effect varies among postgraduates with different registered residence types. Occupational self-efficacy has a more significant role in promoting the career maturity of postgraduates with rural registered residence.

In summary, the level of career maturity not only reflects the employability of postgraduates, but also reflects their future career development, and is an important factor affecting the quality of postgraduate employment. The analysis of the characteristics and influencing mechanism of postgraduates’ career maturity will help postgraduates understand the level of their own career maturity, explore and plan their own career as early as possible, and improve their career decision-making level and career development ability. At the same time, it is also helpful for colleges and universities to provide more targeted employment guidance, improve the efficiency and effectiveness of counseling and guidance, and provide implementation direction for the improvement of postgraduates’ career maturity, so as to help postgraduates alleviate their employment anxiety and achieve high-quality employment.

## Data Availability

The data that support the findings of this study are available from the corresponding author upon reasonable request.
